# Characterization of the Vertical Stiffness Gradient in Cadaveric Human and Excised Canine Larynges

**DOI:** 10.1016/j.jvoice.2024.08.011

**Published:** 2024-09-06

**Authors:** Jacob Michaud-Dorko, Gregory R. Dion, Charles Farbos de Luzan, Ephraim Gutmark, Liran Oren

**Affiliations:** * Department of Biomedical Engineering, University of Cincinnati, Cincinnati, Ohio; † Department of Otolaryngology-Head and Neck Surgery, University of Cincinnati, Cincinnati, Ohio; ‡ Department of Aerospace Engineering, University of Cincinnati, Cincinnati, Ohio.

**Keywords:** Vocal fold elasticity, Young’s modulus, Anterior-posterior vertical stiffness gradient, Micro-indentation

## Abstract

The elastic properties of the folds govern the characteristics of vocal fold vibrations. This study addresses existing gaps by investigating the Young’s modulus along the anterior-posterior direction in excised canine and cadaveric human vocal folds. Micro-indentation testing was conducted on six excised canines and three cadaveric human larynges. Multiple points along the medial glottal wall were indented to determine force-displacement, stress-strain relationships, and Young’s modulus as a function of Green’s strain. A vertical stiffness gradient was consistently observed in both canine and human samples, with higher stiffness in the inferior aspect compared with the superior aspect. The stiffness increased toward both the anterior and posterior directions from the mid-coronal plane, with a more pronounced increase at the posterior edge. Human vocal folds generally exhibited lower stiffness at low strains but were comparable to canine vocal folds at higher strains. These findings suggest that the canine larynx model is a reasonable representation of the human laryngeal tissues’ elastic property trends. This analysis of the vertical stiffness gradient in canine and human vocal folds provides valuable data for improving experimental and numerical models of phonation.

## INTRODUCTION

During speech, air flows from the lungs through the larynx, causing the vocal folds to vibrate and generate sound waves. Key vocal properties, such as pitch and acoustic intensity, are governed by the elastic properties of the vocal folds and regulated by the tension and length of the vocal folds along the anterior-posterior direction.^1^

Characterizing the elastic parameters of the vocal fold, particularly its Young’s and shear modulus, holds promise for refining surgical techniques for vocal fold pathology repair,^2,3^ and enhancing experimental^4^ and numerical^5,6^ models of phonation mechanisms. Various models are commonly employed to study these characteristics, including cadaveric human, excised canine, and porcine specimens. Mechanical testing methods such as longitudinal elongation, linear skin rheometry, and micro-indentation have been utilized for this purpose, with a comprehensive review available by Dembinski et al.^7^

While cadaveric human samples offer the closest approximation to real human tissue, obtaining fresh specimens from healthy young individuals is challenging. Cadaveric human larynges often come from older individuals and exhibit significant tissue atrophy, which impacts measured vocal fold tissue elasticity values. Canine specimens are preferred in voice research due to their anatomical and acoustic similarities to humans.^8^ Additionally, the biomechanical properties can be collected immediately postmortem, minimizing tissue decay and allowing for more ideal measurements. Porcine larynges, which also vibrate at a frequency similar to humans, can be easily obtained, and elastic parameters can be immediately collected postmortem.^9^ However, differences in anatomy, including two sets of oscillating vocal folds, hinder direct comparison to human,^10^ making the canine larynx model a preferred choice for studying vocal fold elastic parameters.

In a study by Oren et al,^11^ Young’s modulus of both the superior and inferior aspects of ex vivo canine larynges was measured using the micro-indentation technique in the mid-coronal plane. The measurements were conducted on 11 excised canine specimens, revealing that the inferior aspect of the vocal fold consistently exhibited greater stiffness than the superior aspect. Moreover, the study demonstrated that the disparity in stiffness between the inferior and superior aspects increased at higher strain values (ie, displacement), resulting in Young’s modulus difference of 35% at 40% strain. The researchers suggested that this difference in stiffness might contribute to the divergent shape seen in the vocal folds during the closing phases of phonation.

The glottal space changes shape during phonation between the opening and closing phases. In this flow-induced self-oscillatory state (ie, phonation), the vocal folds alternate between a convergent (during the opening phases) and divergent (during the closing phases) glottal shape.^12^ This convergent-divergent glottal shape ultimately controls and affects the glottal airflow dynamics.^13^ The convergent-divergent glottal shape produces a phase delay between the lower and upper edges of the glottis (ie, vocal folds), known as the vertical phase delay, and can be observed through videostroboscopy.^14^

Using linear stability analysis, it was shown that a convergent-divergent shape is necessary for vibration to occur.^15^ In addition, without the presence of a mucosal wave, it was shown that the convergent-divergent geometry occurs because of a transfer of aerodynamic energy from the glottal flow to the movement of the folds.^16^ Experiments, particularly those using indentation testing on human and canine larynges, as previously discussed, have shed light on the vertical stiffness gradient, revealing its significant role in the vertical phase delay.^17,18,11^ Although much about voice mechanisms and disorders remains unknown, altering vocal fold elastic parameters results in vocal pathology, potentially requiring surgical treatment. This was shown by the removal of the vertical stiffness gradient and its impact on acoustic properties, further emphasizing its importance in healthy phonation dynamics and acoustic output.^2^

Most studies on the vocal fold vibration mechanism have focused on the mid-coronal plane, assuming it reflects the overall glottal behavior. Studies of laryngeal flow have been performed by approximating the glottal opening as a two-dimensional geometry during numerical simulations or only collecting flow field measurements in the mid-coronal plane of experimental models. However, researchers have highlighted the importance of considering the three-dimensional nature of the intraglottal and supraglottal jet.^19–22^ Steady flow investigations through three-dimensional static glottal orifices have reported secondary flows in off-center coronal sections with velocity magnitudes as high as 10% of the axial flow velocities.^23^ This velocity component is driven by the anterior-posterior pressure gradient neglected in two-dimensional flow investigations. Supraglottal flow in excised canine larynges also exhibits significant velocity gradients in the anterior-posterior direction, shown by axis-switching of the glottal jet orientation.^24^ Furthermore, mechanical indentation testing on ex vivo excised canine vocal folds, conducted along the anterior-posterior direction, revealed lower stiffness values along the superior aspect of the vocal fold compared with the inferior aspect.^25^ Notably, the lowest tissue stiffness was observed at the mid-coronal location, with stiffness increasing in the anterior direction and even more so in the posterior direction. This discovery offers further evidence to explain the presence of an anterior-posterior pressure gradient in the flow field, likely attributable to the variation in the vertical stiffness gradient across anterior-posterior locations.

One of the many objectives of voice research is to establish a model capable of enhancing surgical outcomes. Whether numerical or experimental, this model could serve as a guide for optimal interventions for individuals with voice disorders. Such advancements will offer clinicians innovative tools to evaluate patients, perform surgical planning, and deepen our comprehension of voice disorders through a methodical procedure.

To bridge the knowledge gap concerning vocal fold tissue elastic properties and to provide additional insights for experimental and numerical models as a potential surgical aid, a thorough understanding of the vertical stiffness gradient along the anterior-posterior direction in excised larynges is imperative. Currently, only one study has analyzed the anterior-posterior vertical stiffness gradient in excised canine larynges.^25^ The limitations of this study are the spatial resolution of the data points collected along the vocal fold, the speed at which the load cell was applied to the tissue, and only the collection of the loading portion was used for the force and stiffness measurements. Fourteen data points were collected along the vocal fold (seven inferior and seven superior), and 25 additional points (four rows) were collected in the subglottis, all along the anterior-posterior direction. Additionally, the indentation speed was 6 mm/s, which may have caused peaks in the normal force vs displacement curve due to inertia effects, potentially leading to increased force measurements. Moreover, only the loading portion of the force measurement was reported and used for stiffness analysis. Previous studies have shown that unloading force measurements should minimize measurement artifacts caused by global tissue displacement.^26^

The present study aims to address these limitations by increasing the spatial resolution of tissue elasticity measurements along the anterior-posterior direction in excised canine and cadaveric human vocal folds. Micro-indentation testing was conducted to determine force-displacement, stress-strain relationships, and Young’s modulus as a function of Green’s strain. Data collection was performed at 0.2 mm/s to reduce the inertia effect on the tissue, and the unloading curve was subsequently used for tissue stiffness analysis.

## METHODOLOGY

**FIGURE 1.**
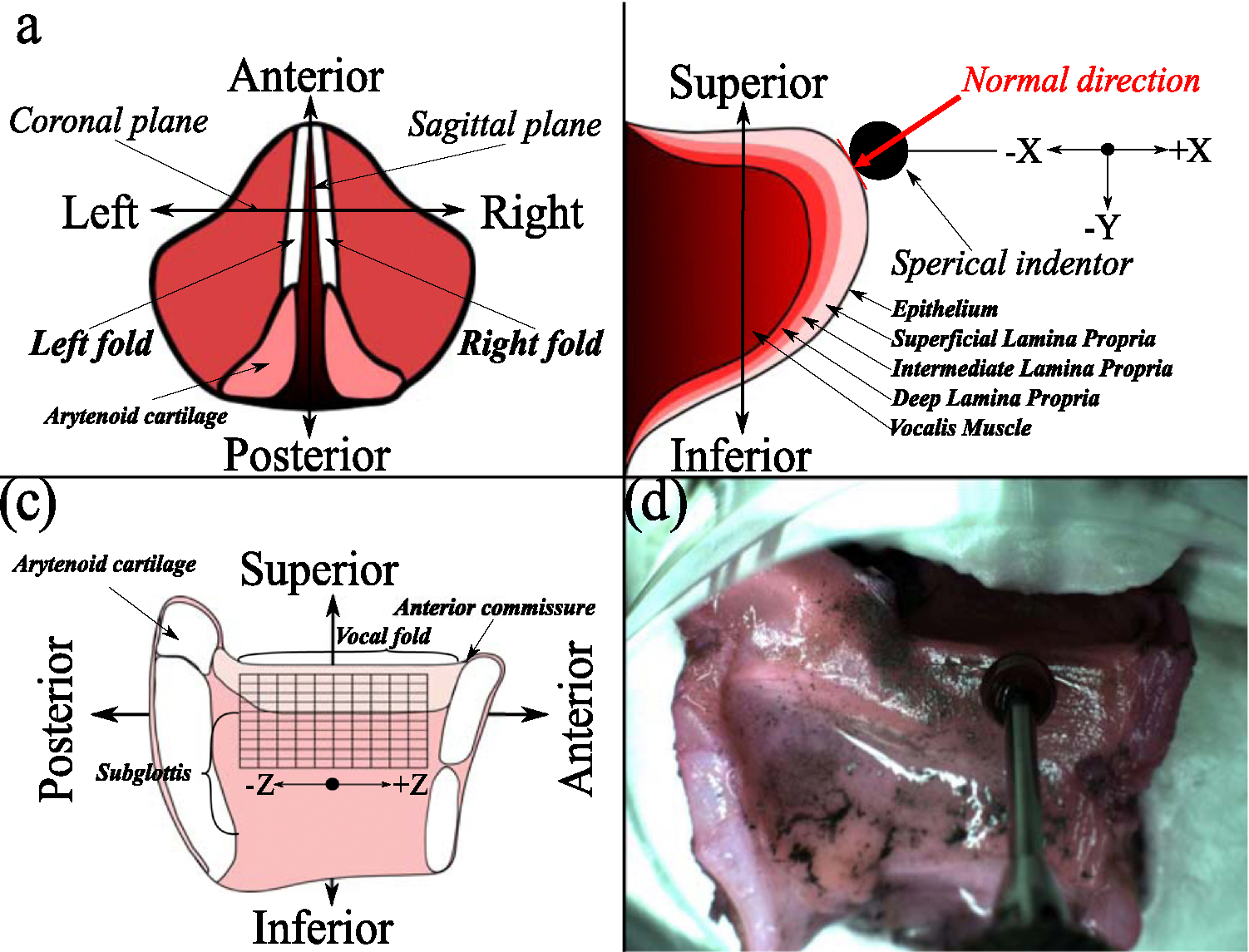
Experimental setup for indentation mapping of an excised canine larynx. (a) Transverse view of the larynx for reader orientation. (b) Coronal view of the left vocal fold showing the vocal fold layers and the normal indentation process. (c) A sagittal view of the left vocal fold illustrates where force data were collected during indentation mapping. (d) The excised canine vocal fold was positioned in a specimen holder and oriented under the spherical indenter before data collection (not shown in phosphate-buffered saline).

The elastic properties of the vocal folds for each larynx were collected following the procedure outlined by Dion et al.^25^ Six excised canines (CL1-CL6) and three cadaveric human larynges (HL1-HL3) were prepared by removing all cartilage and soft tissue above the vocal folds and below the first tracheal ring. The larynges were bisected in the sagittal plane, cutting through the center of the anterior commissure to create a hemilarynx configuration. Figure 1a is provided to orient the reader to the viewing perspective used throughout this paper.

The hemilarynges were stored in saline in a refrigerator at 3.3°C until testing. Before testing, each hemilarynx was brought to room temperature. To secure the hemilarynges during testing, custom sample holders were created for each specific vocal fold (canine and human). These holders were made from plaster and fitted to each hemilarynx specimen. After drying, the plaster was protected by plastic wrap and coated with a protective water-based varnish. The hemilarynx samples were fixed to the plaster holder using cyanoacrylate adhesive and tested in phosphate-buffered saline (PBS) to maintain moisture. Samples were equilibrated for 15 minutes in PBS prior to indentation testing.

The gender and exact age of the human cadavers were unknown, but it should be noted that each human larynx was excised from senior citizens with no known comorbidities. Similarly, the age and gender of the excised canine larynges were also unknown; however, each canine weighed between 45 lbs and 47 lbs with no known comorbidities. The length of the vocal folds ranged from 8 mm to 9 mm for humans and from 13 mm to 14 mm for canines. The reported average height of the human vocal folds is approximately 2.3 mm (± 0.4 mm),^27^ while the canine vocal fold measures slightly more, averaging 2.5 mm (± 0.5 mm).^11^ Despite differences in the overall length of the vocal folds between species, the similarity in height strengthens the argument for using canine larynges as a representative model.

A Biomomentum Mach-1 (Biomomentum Inc., Laval, Quebec, Canada) mechanical tester with a 150-g single-axis load cell and a 7.5-mg resolution performed automated indentations on the nonplanar hemilaryngeal surfaces. For each vocal fold specimen (left and right), the vocal folds were indented 2 mm perpendicular to the surface using a spherical indenter with a radius of 2.5 mm at each specific data point (Figure 1b). A normal indentation of 2 mm was chosen since typical glottal width for excised canines is between 3 mm and 4 mm for low and high subglottal pressure, respectively.^28^

The slope of the surface of each vocal fold was determined by probing the spherical indenter in four locations around the data collection spot to identify the gradient of the surface. The probe was then indented normal to this surface gradient at the specified measurement location at a speed of 0.2 mm/s, held for 1 second at maximum displacement, and then unloaded at the same speed. The load cell data were sampled at 1 kHz throughout the indentation test (ie, loading, pause, and unloading).

This process was repeated in a random order across all specific data collection points on each vocal fold in the superior-inferior direction and along the length of the vocal fold in the anterior-posterior direction (Figure 1c). The collected data points formed a grid 10 mm in length and 5 mm in height for the canine larynx vocal folds, with 1-mm steps along the anterior-posterior direction and 0.5-mm steps along the superior-inferior direction, resulting in a total of 121 data points. For the human larynx vocal fold, the grid was 6 mm in length and 5 mm in height, with 1-mm steps in the anterior-posterior direction and 0.5-mm steps along the superior-inferior direction, totaling 77 data points. A schematic for the data points collected for the canine larynx model is shown in Figure 1c, where the intersection points indicate the approximate locations of the force measurements taken when indenting the spherical probe 2 mm normal to the surface.

The grid data points were initially collected just below the superior aspect of the vocal fold to ensure that the spherical indenter did not slip off the edge of the vocal fold and to achieve a more uniform contact area between the tissue and the spherical probe. Additionally, the 2.5-mm radius of the spherical indenter restricted its proximity to the anterior commissure. There was a concern that the indenter might contact the cartilage, preventing accurate tissue elasticity data collection. Figure 1d shows an image of an excised canine larynx under the spherical indenter connected to the load cell, mounted in a sample holder made for that specific larynx. Note that the excised canine tissue in Figure 1d is not shown in PBS to provide a clear image of the experimental setup used for data collection in this study.

The tissue stress was calculated following the stress equation: σ = F/A_contact_, where F is the force measurements from the load cell and A_contact_ is the contact area between the tissue and the spherical probe. The contact area was determined using the spherical cap equation: A_contact_ = 2πrh, where r is the radius of the spherical indenter, and h is the indentation displacement with respect to the datum, starting at the tangent to the contact surface location, as shown in Figure 1b. The uniform strain in the tissue was calculated using the Green’s strain equation: ${\text{ε}}_{\text{G}}=\left({\text{L}}_{\text{o}}^{2}-{\text{L}}^{2}\right)/2{\text{L}}_{\text{o}}^{2}$, where $L_{o}$ is the undeformed width of the tissue and L is the deformed width. The Young’s modulus of the tissue (ie, stiffness) was determined by the derivative of the stress-strain curve: $E=\partial \sigma /\partial {\text{ε}}_{\text{G}}$, where σ is the stress, and ${\text{ε}}_{\text{G}}$ is Green’s strain.

The reason for implementing the methodological procedure outlined by Dion et al^25^ is its potential to provide more accurate indentation data due to using a spherical probe on a nonplanar surface. Previous work has employed plane-ended cylindrical indenters with a radius of 0.5 mm for normal indentation depths greater than 0.5 mm.^29,17,18,11^ Although frequently used in the past, this method has more associated limitations compared with using a spherical indenter.^30^

Due to its radius of curvature, a spherical indenter allows for better alignment to indent normal to the surface, leading to a more uniform distribution of stress between the tissue and the probe. This reduces shear stress buildup and prevents tissue from curling over the edges of the measurement surface if the indentation depth does not exceed the radius of the spherical probe. Consequently, this method provides more accurate measurements of normal stress in the tissue per unit area.

## RESULTS

**FIGURE 2.**
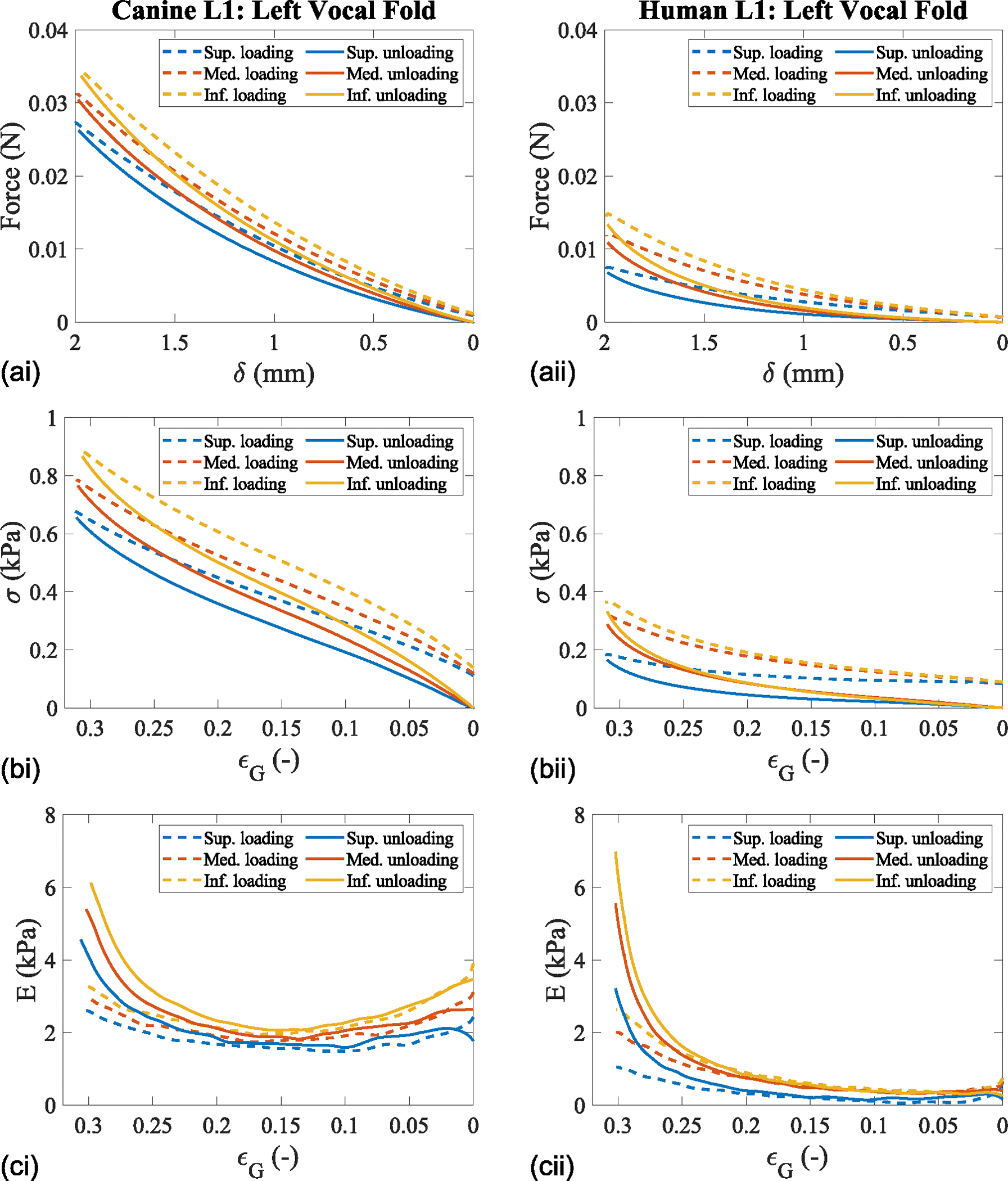
Mid-coronal indentation measurements in excised canine and human vocal folds. (a) Force versus displacement. (b) Stress versus strain. (c) Young’s modulus versus Green’s strain. Left column—canine L1. Right column—human L1.

**FIGURE 3.**
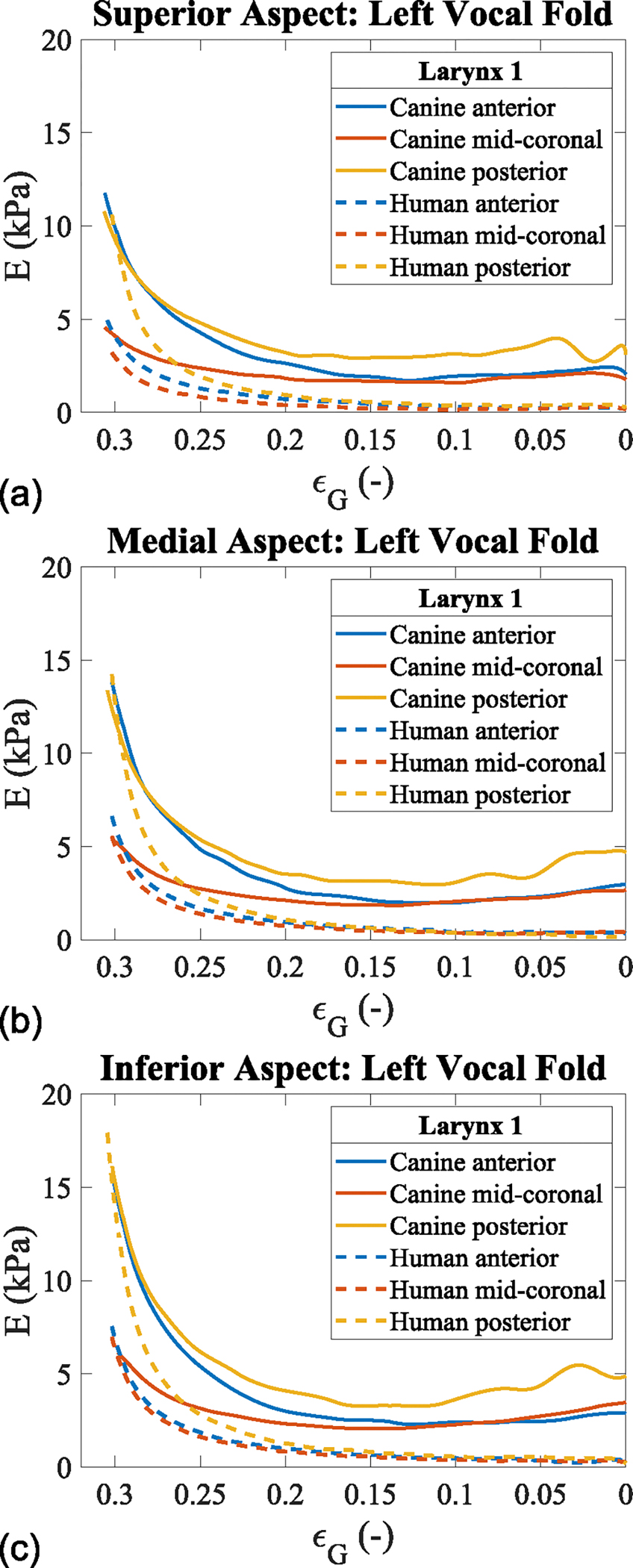
Anterior-posterior unloading Young’s modulus versus Green’s strain in excised tissue models. (a) Superior aspect (Y = 0 mm). (b) Medial aspect (Y = −1 mm). (c) Inferior aspect (Y = −2 mm).

**FIGURE 4.**
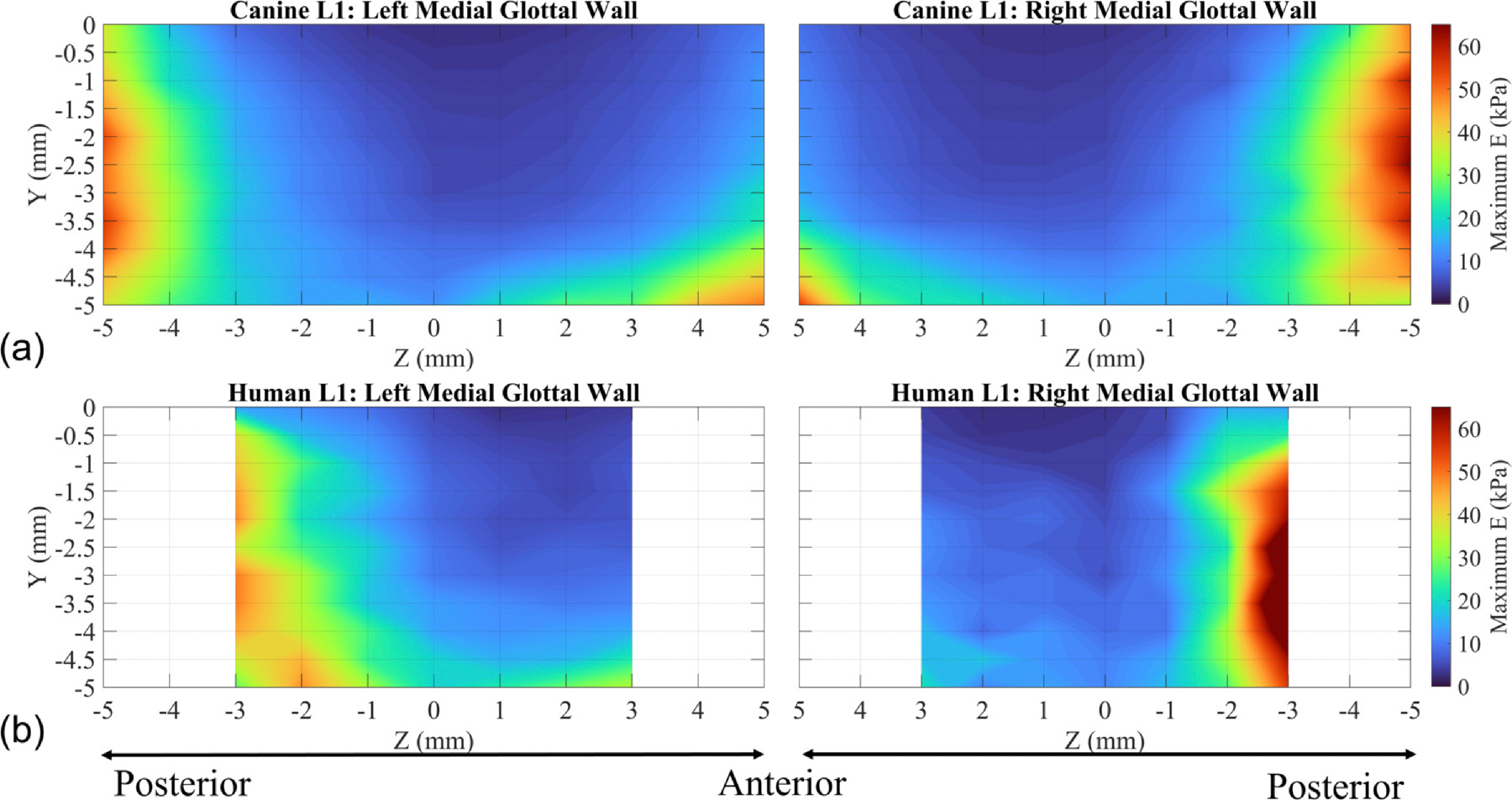
Maximum Young’s modulus along the medial glottal wall. (a) Excised canine larynx 1. (b) Cadaveric human larynx 1.

**FIGURE 5.**
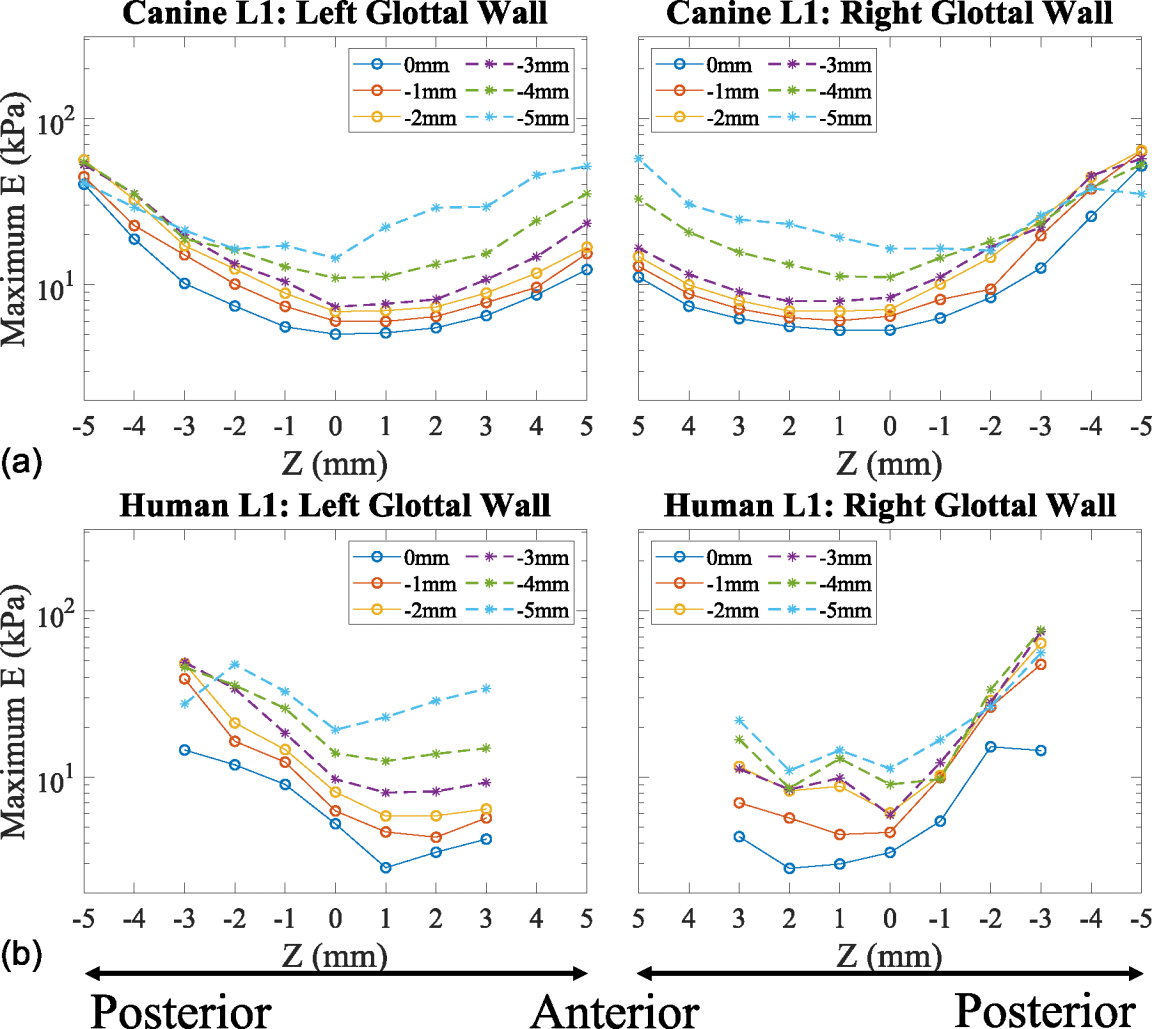
Maximum Young’s modulus profile along the medial glottal wall. (a) Excised canine larynx 1. (b) Cadaveric human larynx 1.

**FIGURE 6.**
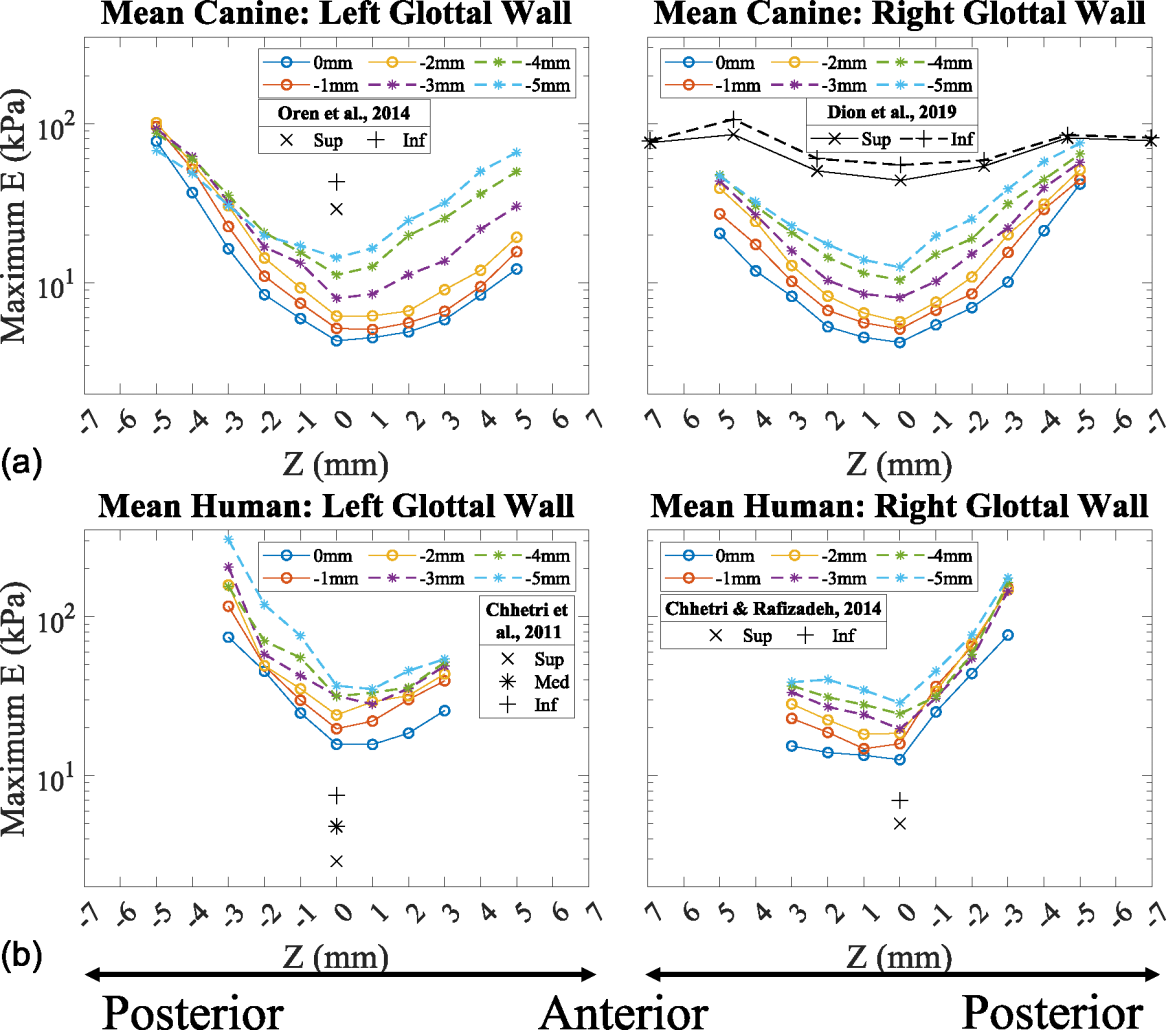
Mean maximum Young’s modulus profiles along the anterior-posterior direction. (a) Excised canine. (b) Cadaveric human.

**FIGURE 7.**
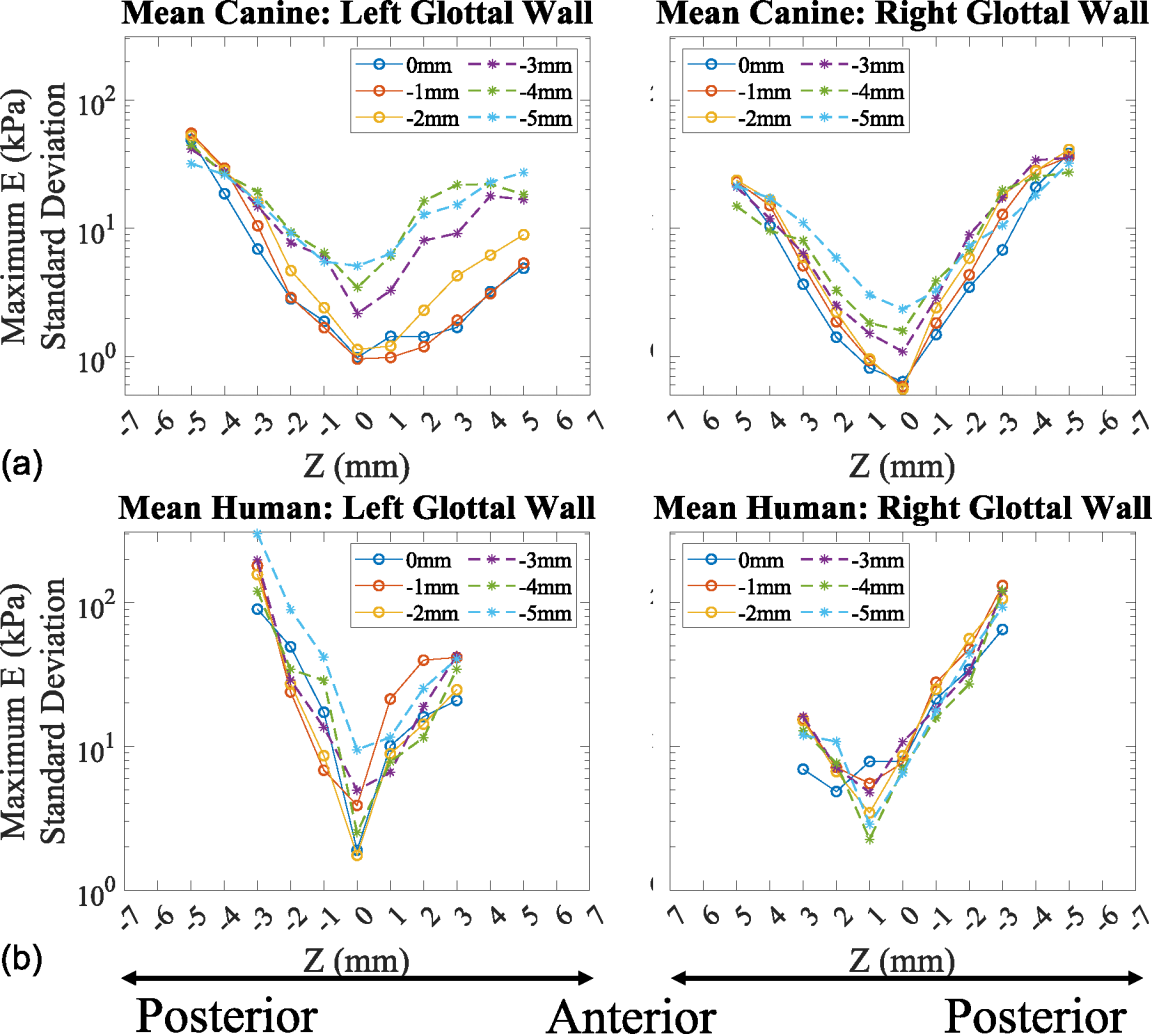
Standard deviation of maximum Young’s modulus profiles along the anterior-posterior direction. (a) Excised canine. (b) Cadaveric human.

As the data measured in the left and right vocal folds were consistently nearly symmetrical across all larynges, the dissemination of the results in Figures 2 and 3 is streamlined by comparing the measurement data from one vocal fold from a canine larynx and one vocal fold from a human larynx, namely the left one. The near-identical measurements of the left and right vocal folds in all larynges are further illustrated in Figures 4 and 5 by comparing the measurement data from one canine and human larynx. The combined data from all larynges are shown in Figure 6 with the corresponding standard deviation shown in Figure 7.

The steps to calculate the force, stress, strain, and Young’s modulus in this study are first shown in Figure 2. The left column presents the findings for the canine (CL1) and the right column presents the findings for the human (HL1) vocal fold. The loading and unloading curves for one test are shown for the left vocal fold’s superior, medial, and inferior locations in the mid-coronal plane. The dashed line represents the loading portion, and the solid lines represent the unloading portion of data collection. The force-displacement data were smoothed using a moving mean filter with a window of 0.1 mm (Figure 2a). This force-displacement data were used for the subsequent calculations of stress (Figure 2b) and Young’s modulus (Figure 2c) of the excised tissue vocal folds and subglottis.

Hysteresis, indicated by the differences between the loading and unloading curves of the force and stress measurements, is observed in both models (Figure 2a and b). The canine vocal fold demonstrates higher force and stress values than the human vocal fold, potentially due to histological differences and less tissue decay.

Following the stress-strain analysis, both tissue models display a vertical stiffness gradient during loading and unloading (Figure 2c). Interestingly, at ε_G_ = 0, the Young’s modulus of the canine vocal fold are on average 130% and 164% higher for the loading and unloading curves, respectively, compared with the human vocal fold. Furthermore, the stiffness as a function of Green’s strain shows a parabolic curve from low to maximum strain for the canine vocal fold, whereas the human vocal fold exhibits a half-parabolic curve.

After the tissue was indented to a maximum depth of 2 mm, corresponding to ε_G_ = 0.32, and held for 1 second before unloading, Young’s modulus was measured for both canine and human vocal folds (Figure 2c). The maximum unloading Young’s modulus values for the superior, medial, and inferior locations of the vocal folds in the mid-coronal plane were 4.6 kPa, 5.4 kPa, and 6.0 kPa for the canine vocal fold, and 3.2 kPa, 5.5 kPa, and 7.0 kPa for the human vocal fold, respectively.

The remainder of the results for the current study data will focus on the unloading Young’s modulus, as it minimizes measurement artifacts caused by global tissue displacement and to observe the viscoelastic properties—specifically, the stress relaxation.^26^ The unloading Young’s modulus-displacement curves are shown for the superior, medial, and inferior locations along the anterior-posterior direction for the left excised canine (CL1) and human vocal folds (HL1) (Figure 3). In these graphs, the superior, medial, and inferior locations are represented at Y = 0, −1, and −2 mm, respectively. For the canine vocal fold, the anterior, mid-coronal, and posterior locations are shown at X = 5, 1, and −3 mm, while for the human vocal fold, they are at X = 3, 1, and −1 mm, respectively. These specific points were selected to illustrate the vertical stiffness gradient observed at the furthest anterior point of the collected data, the mid-coronal plane, and the posterior plane before the vocal processes. These locations were chosen because they display the stiffness gradient in regions where vibration occurs, which is why the location of the vocal processes was not included, as little-to-no vibration occurs there during phonation.

A vertical stiffness gradient is observed in the vocal folds’ posterior, mid-coronal, and anterior locations for both excised tissue models. As normal displacement increases, the vertical stiffness gradient also increases, reaching its maximum at a displacement of 2 mm or ε_G_ = 0.32.

In both tissue models, the stiffness gradient along the medial glottal wall is evident, with Young’s modulus values increasing from the superior to the inferior aspects of the vocal fold (Figure 3). This vertical stiffness gradient is consistent in both the anterior and posterior directions relative to the mid-coronal plane. In the posterior direction, the maximum Young’s modulus values increased by 57.0%, 59.7%, and 62.3% for the superior, medial, and inferior locations of the canine vocal fold, and by 69.8%, 60.7%, and 60.9% for the human vocal fold, with respect to the mid-coronal location. Similarly, in the anterior direction, the increases were 61.0%, 60.9%, and 61.0% for the superior, medial, and inferior locations on the canine vocal fold, and 34.7%, 15.4%, and 6.7% for the human vocal fold, with respect to the mid-coronal location.

Overall, the canine vocal fold displayed similar stiffness increases along the anterior-posterior direction, while the human vocal fold showed a larger increase in tissue stiffness at the posterior edge and smaller increases toward the anterior edge. The mid-coronal and posterior locations of both tissue models exhibited similar Young’s modulus values along the vertical height of the vocal folds. However, the anterior direction revealed larger Young’s modulus values in the canine vocal folds compared with the human vocal folds.

Interestingly, the unloading Young’s modulus followed a parabolic curve from low to maximum strain in the anterior-posterior direction for the canine vocal fold, whereas the human vocal fold exhibited a half-parabolic curve. In summary, for both models, a vertical stiffness gradient is observed, with lower Young’s modulus values at the superior aspect of the vocal fold and higher values at the inferior aspect. The stiffness at the inferior, medial, and superior locations all increase proportionally to the distance from the mid-coronal plane.

The stiffness distribution along the glottal wall is shown using contour plots of Young’s modulus values at the maximum indentation depths (Figure 4). These plots further illustrate how the tissue becomes stiffer in the posterior direction as you move away from the mid-coronal plane with only a slight increase in stiffness toward the anterior direction. There is little-to-no vertical stiffness gradient at the furthest posterior side of both the left and right glottal walls, just below Y = −1 mm for both canine and humans. However, a vertical stiffness gradient can be observed approximately 2 mm away from the furthest posterior measurement location (Z = −3 mm for canine and Z = −1 mm for human), continuing toward the anterior edge. The region with the highest Young’s modulus at the posterior edge is due to the presence of the vocal process behind the soft tissue, which is composed of cartilage. The high stiffness value with minimal gradient is often the reason why this part of the vocal fold does not vibrate during phonation.

The maximum Young’s modulus profiles were extracted from the canine and human larynges along the anterior-posterior direction. The data are shown along the vertical height of the medial glottal wall starting from the superior aspect at Y = 0 mm, moving down into the subglottis, and ending at Y = −5 mm (Figure 5). Solid lines with circles (o) represent the vocal fold, while dashed lines with asterisks (*) represent the subglottis.

Overall, in both excised tissue larynges, the left and right vocal folds and the subglottis exhibit a vertical stiffness gradient extending down to Y = −5 mm. This vertical stiffness gradient along the medial glottal wall shows similar trends in canine and human tissues. The stiffness profiles further indicate that the excised canine hemilarynges display stiffness symmetry along the sagittal plane. In contrast, the excised human hemilarynges do not exhibit as pronounced symmetry along the sagittal plane, although both vocal folds display similar vertical stiffness gradient trends.

To determine the overall trends in the observed vertical stiffness gradient along the medial glottal wall, the Young’s modulus for the entire dataset (comprising six canine and three human larynges) was averaged for both the left and right vocal folds (Figure 6). The averaged data show the overall vertical stiffness gradient trends with lower Young’s modulus values at the superior aspect of the vocal fold and higher values at the inferior aspect. The stiffness along the vertical height of the vocal folds all increase proportionally to the distance from the mid-coronal plane, as previously discussed.

The averaged maximum Young’s modulus standard deviation across the vocal folds and into the subglottis for both canine and human larynges is presented in Figure 7. The standard deviation of the maximum Young’s modulus profiles shows that lower levels of variance are observed at the mid-coronal location (Z = 0 mm) and increase toward the anterior and posterior direction in both the canine and human tissue models.

## DISCUSSION AND CONCLUSION

The main similarities and differences between human and canine vocal folds are as follows: human and canine vocal folds exhibit a vertical stiffness gradient, with higher stiffness in the inferior aspect compared with the superior aspect. Canine vocal folds generally exhibit higher force values and stiffness than human vocal folds, potentially due to the closer proximity of the conus elasticus to the surface of the canine vocal fold and the longer tissue decay in the human samples used in this study. Young’s modulus values increase proportionally toward both models’ anterior and posterior directions from the mid-coronal plane. Still, this increase is more pronounced at the posterior edge of human vocal folds. Both canine and human vocal folds exhibit similar trends in the vertical stiffness gradient and anterior-posterior stiffness variations, suggesting that canine models can reasonably represent the trends in elastic properties of the human vocal folds.

Previous research has documented that the elastic properties of vocal folds significantly affect parameters such as pitch, acoustic intensity, and intraglottal airflow during phonation. However, there are gaps in our understanding, particularly regarding the elastic properties along the anterior-posterior direction of the medial glottal wall. While excised human samples provide valuable data, their limited availability has led researchers to use the canine larynx model. Notably, most studies have focused on the mid-coronal plane, potentially overlooking significant variations in the anterior-posterior direction. This study addresses these gaps by increasing the spatial resolution of stiffness measurements along the anterior-posterior direction in excised canine and human vocal folds.

Our experiments revealed crucial insights into the elastic properties of vocal folds. A vertical stiffness gradient was observed in canine and human samples, with the inferior aspect of the vocal fold showing higher stiffness than the superior aspect. This gradient was consistent across different anterior-posterior locations, with stiffness increasing proportionally to the distance from the mid-coronal plane, with a slightly greater increase at the posterior edge.

Compared with other studies, our findings from the canine hemilarynges align with previous research by Oren et al,^11^ which reported higher stiffness in the inferior aspect of the vocal folds than the superior aspect. However, our study extends this work by providing detailed data along the anterior-posterior direction of the excised canine medial glottal wall, offering a more comprehensive view of the stiffness gradient. Our results also support the observations of Dion et al,^25^ who found variations in stiffness along the anterior-posterior direction and higher stiffness in the inferior aspect of the vocal folds. Our data confirm these findings and enhance the spatial resolution, providing a finer map of the elastic parameters along the medial glottal wall.

The unloading stress curves in the superior and inferior locations qualitatively agree with the measurements by Oren et al.^11^ However, their measurements, collected with a flat cylinder probe, are known to increase the collected stress measurement due to a shearing effect on the tissue, resulting in values roughly 1.5 to 1.75 times higher than those measured in this study.

Using a planar surface for normal indentation measurement increases shear stress in the tissue due to the edges of the surface. This leads to higher stress and Young’s modulus values, as the collected data include normal and shear stress. Given the nonplanar nature of the tissue surface, achieving normal indentation with a planar surface is difficult, further increasing shear stress and skewing the data.

Additionally, a planar surface probe causes the tissue to curl over the edges of the measurement surface, resulting in non-normal contact with the probe and further skewing the data to higher values. Therefore, using a spherical probe for normal indentation measurements offers the most reliable and accurate collection of normal force in the tissue when a pressure load is applied.

A plane-ended cylindrical indenter can be utilized when deformation is directly proportional to the applied load. This confines its use to scenarios involving small stress-strain values and low indentation depths that do not exceed the radius of the planar surface.

When comparing the inferior and superior stiffness values for the collected mean dataset in the present study to Oren et al^11^ and Dion et al,^25^ our data show lower mean stiffness values in the mid-coronal plane and along the anterior-posterior direction. An average difference of 162% and 163% was observed in the mid-coronal location between the present study and the work published by Dion et al^11^ and Oren et al,^25^ respectively. The higher stiffness values reported by Oren et al^11^ are a result of the increased shear stress previously discussed. The higher values reported by Dion et al^25^ could be due to the faster indentation speed used in the study and the reported values extracted from the loading portion.

The collected stiffness measurements for the mean sample of human vocal folds had higher stress values than those reported by Chhetri et al.^17,18^ This is because the present study included all tissue structures, such as the cover, ligament, and body layers, in the indentation measurements. In contrast, these previous studies isolated the cover layer of the vocal fold.

While micro-indentation provides detailed measurements of tissue stiffness, it may not fully capture the dynamic behavior of vocal folds during actual phonation. Thus, the results only approximate the potential stress in vocal folds during phonation. Therefore, several limitations must be mentioned. The sample size, particularly for human larynges, was small, which could impact the generalizability of the results. Additionally, atrophy of the human tissue can lower the measured stiffness values, as shown by the low-stress values at low strain values. Whereas canine specimens, usually tested immediately postmortem, are less affected by tissue decay, leading to more accurate measurements of their biomechanical properties. This is evident from the low standard deviation in the canine sample set and the high standard deviation in the human sample set.

The canine vocal fold, as described by Hirano,^31,32^ lacks a well-established ligament, with its superficial layer being notably thicker than that of humans. This histological difference likely has an impact on the vibratory properties of the vocal folds. It has been speculated that this structural difference might explain why canines can initiate phonation but struggle to maintain it, particularly at higher frequencies where longitudinal tension is necessary.^33^ The well-defined ligament in humans may be vital for achieving the fine control necessary to sustain high-pitched sounds, potentially highlighting the critical role of this structural component in human vocal function.

During the sagittal transection of the larynges for indentation measurements, Broyle’s ligament was kept intact with the thyroid cartilage. By preserving the integrity of Broyle’s ligament to the cartilage, the focus was to maintain the natural anatomical relationships and stiffness characteristics as much as possible. However, this procedure might have altered the stiffness on the anterior side of the vocal folds.

The insights gained from the current study open multiple avenues for future research. One significant direction is exploring how the observed stiffness gradient in the anterior-posterior directs phonatory dynamics and acoustic properties. More sophisticated computational models that incorporate three-dimensional stiffness data are also needed to better simulate the complex fluid-structure interaction of vocal folds during phonation. These advancements could improve surgical techniques and deepen our comprehension of voice disorders.

## Data Availability

The data that support the finding of this study are available from the corresponding author upon request.
